# Review of the Genus *Sciara* Meigen, 1803 (Diptera, Sciaridae) in Ukraine

**DOI:** 10.3390/insects14090732

**Published:** 2023-08-30

**Authors:** Andriy Babytskiy, Serhii Pavliuk, Olesia Bezsmertna

**Affiliations:** 1 Department of Entomology and Collection Management, Schmalhausen Institute of Zoology NAS of Ukraine, vul. Bohdana Khmelnytskogo, 15, 01030 Kyiv, Ukraine; 2 Department of Agrosphere Ecology and Environmental Control, National University of Life and Environmental Sciences of Ukraine, vul. Heroiv Oborony, 13, 03041 Kyiv, Ukraine; pavlyuksd@ukr.net; 3 Department of Ecology and Zoology, Taras Shevchenko National University of Kyiv, vul. Volodymyrska, 60, 01033 Kyiv, Ukraine; olesya.bezsmertna@gmail.com; 4 Tsumanska Puscha National Nature Park, vul. Nezalezhnosti, 18, Kivertsi Town, 45200 Kyiv, Ukraine

**Keywords:** Sciaridae, black fungus gnats, faunistics, species diversity, morphology, Europe

## Abstract

**Simple Summary:**

Sciarids (Diptera, Sciaridae) are small, mostly dark-colored insects whose larvae usually develop in decaying remains of plants that are penetrated by fungal hyphae. Typical habitats for sciarids are shady forests and wet meadows, but some species can migrate from natural biotopes to anthropogenic ecosystems and live as synanthropes. The genus *Sciara* Meigen, 1803 is an original genus of the Sciaridae family and includes 55 valid *Sciara* species in the world fauna. *Sciara* species play an important role as detritivores, facilitating the decomposition of decaying wood and leaf litter in biotopes. In the paper, the diversity of *Sciara* in Ukraine is analyzed with some remarks on their ecology, phenology and distribution.

**Abstract:**

On the territory of Ukraine during the field seasons 2015–2022, we collected eight *Sciara* species—*S. analis* Schiner, 1864, *S. flavimana* Zetterstedt, 1851, *S. hebes* (Loew, 1869), *S. helvola* Winnertz, 1867, *S. hemerobioides* (Scopoli, 1763), *S. humeralis* Zetterstedt, 1851, *S. incerta* Winnertz, 1867 and *S. ruficauda* Meigen, 1818. All specimens are kept in the collections of the Schmalhausen Institute of Zoology NAS of Ukraine (SIZK). According to the results of our research, the four species *S. flavimana*, *S. hebes*, *S. helvola* and *S. incerta* are being registered for the first time in Ukraine. New localities are given for *S. analis*, *S. hemerobioides*, *S. humeralis* and *S. ruficauda*. The distribution, morphological peculiarities, ecology and phenology of registered species are also briefly discussed. A key to 12 European *Sciara* species is given.

## 1. Introduction

*Sciara* Meigen, 1803 is a phylogenetically ancient genus of the family Sciaridae and includes the earliest described sciarid species, which is obviously related to their remarkable appearance, especially their large size (up to 6.7 mm in length for adult males and 8.1 mm for females). In addition, adult females of some completely black species (*S. analis*, *S. hemerobioides*) have broad bright yellow or orange lateral bands on both sides of the abdomen. The larvae of some *Sciara* (e.g., *S. militaris*) can also be found migrating in processions of thousands of individuals and up to 10 meters long. These processions form a group that resembles a snake or a large worm, the so-called “armyworm” (in Europe) and “snakeworm” (in North America). These morphological and developmental peculiarities make *Sciara* one of the most remarkable genera of the Sciaridae.

*Sciara* species usually occupy shady forests and wet meadows, where their larvae feed on plant detritus that is penetrated by the hyphae of the fungus. These species play an important role as detritivores, facilitating the decomposition of decaying wood and leaf litter in biotopes. Under favorable conditions, larvae of *Sciara* species often develop in great masses and contribute to the rapid physical destruction of plant remains in the litter [1].

There is little information on the food preferences of *Sciara* species and their trophic relationships. *Sciara ruficauda* has been recorded as xylophagous and its larvae, ready for pupation, have been found on the surface of the fruit body of polypore fungi [2]. Adult males of *S. militaris* were reared from larch wood, *S. hemerobiodes* from rotten wood and litter [3] and *S. hebes* from a bird’s nest and a meadow pipit’s nest [4,5]. *Sciara incerta* was collected from the inside of a wasp nest in a pine stump [2].

There are 55 valid *Sciara* species known in the world’s fauna [6]. The center of *Sciara* distribution is the Neotropical realm, and in the Palearctic, only about 20 species are known [7]. In Europe, 12 *Sciara* species in three species groups are divided morphologically: *ruficauda* group—*S. flavimana* Zetterstedt, 1851, *S. melanostyla* Mohrig and Krivosheina, 1990 and *S. ruficauda* Meigen, 1818; *humeralis* group—*S. humeralis* Zetterstedt, 1851 and *S. bryophila* Menzel, Salmela and Vilkamaa, 2020; *hemerobioides* group—*S. analis* Schiner, 1864, *S. hebes* (Loew, 1869), *S. helvola* Winnertz, 1867, *S. hemerobioides* (Scopoli, 1763), *S. incerta* Winnertz, 1867, *S. lackschewitzi* (Lengersdorf, 1934) and *S. militaris* Nowicki, 1868 [7,8,9].

The sciarid fauna of Ukraine remains poorly studied [10,11]. The current number of registered Sciaridae in Ukraine contains 101 species from 17 genera [12]. This list includes only five *Sciara* species [13].

The first records of *Sciara* on the territory of Ukraine belong to the second half of the XIX century and show two species (*S. analis* and *S. hemerobioides*) in Galicia, the western part of the modern Ukrainian territory [14,15]. Another species, *S. militaris,* is recorded in the modern Lviv and Ivano-Frankivsk regions after the registration of the “armyworm” phenomenon, caused by its larvae [16,17].

Later, Gerbachevskaja showed four *Sciara* species for Crimea—*S. analis*, *S. hemerobioides*, *S. ruficauda* and *S. humeralis* [18]. Krivosheina and Mohrig reported the Zakarpatska region as a new locality for *S. hemerobioides* [19]. Thus, according to the literature, five *Sciara* species have been recorded in four regions of the modern territory of Ukraine.

There is a lack of comprehensive data on the economic importance of *Sciara* [20]. There are only fragmentary reports in the literature on its damaging effect on crops or mushroom cultivation. *S. hemerobioides* is reported to be a vector of *Claviceps* Tul., 1853 spores (the ergot fungi infect grass or cereals and can cause ergotism—a severe disease in humans or animals) [21,22]. Similar behavior is reported for *S. humeralis* [2].

In this study, we report the findings of eight *Sciara* species in Ukraine: *S. analis* Schiner, 1864, *S. flavimana* Zetterstedt, 1851, *S. hebes* (Loew, 1869), *S. helvola* Winnertz, 1867, *S. hemerobioides* (Scopoli, 1763), *S. humeralis* Zetterstedt, 1851, *S. incerta* Winnertz, 1867 and *S. ruficauda* Meigen, 1818. Basically, the distribution in the world and in Ukraine, morphological peculiarities, habitat preferences and phenology of these species are analyzed.

## 2. Materials and Methods

The material for this study was collected during expeditions and excursions carried out in the field seasons from 2015 to 2022. Adult black fungus gnats were collected with a sweepnet or an aspirator. Collected gnats were stored in 5 mL vials containing 70% ethanol. During laboratory preparation, male specimens were dehydrated in absolute ethanol and then mounted on slides in Euparal.

The specimens were examined with microscopes MBS-9 and PZO Biolar. Photographs of specimens mounted on slides were taken in the Center of collective use of scientific instruments “Animalia” of the Schmalhausen Institute of Zoology NAS of Ukraine using a Zeiss Axio Imager M1 microscope equipped with a digital imaging system. Images were stacked using Helicon Focus 6.7.1 and processed using Photoshop CC 2018. Measurements were made using a PZO Biolar microscope equipped with Nikon D90 cameras and AxioVision40 Version 4.6.3 open-source software.

All of the material examined in this study is held in the funds of Schmalhausen Institute of Zoology NAS of Ukraine, Kyiv (SIZK), as the Sciaridae collection, and publicly available data are on the GBIF [23]. Individual catalogue numbers are given for all of the specimens (e.g., No. 45). The morphological terminology follows the handbook of Palaearctic sciarids by Menzel and Mohrig (1997) [24]. Diagnoses of the discussed species are generally based on examined specimens from Ukraine, considering keys, original descriptions and re-descriptions by Loew (1869) [25], Lengersdorf (1928–1930) [26], Antonova (1978) [2], Mohrig, Krivosheina and Mamaev (1983) [27], Freeman (1983) [4] and Menzel and Mohrig (2000) [7]. Taxonomy and nomenclature follow Menzel and Mohrig (1998, 2000) [7,28] and Mohrig et al. (2013) [29]. Habitat types follow the “National habitat catalog of Ukraine” (2018) [30] in accordance with the biotope classification of the European Nature Information System (EUNIS, 2023) [31].

## 3. Results

### 3.1. Genera Diagnosis

*Sciara* is clearly separated from the other Sciaridae genera by its morphological characters. Head roundish. Ocelli in a flattened triangle behind the eye bridge. Eye bridge consisting of three–four facet rows (rarely with two rows). Palpi long, three-segmented, basal segment without sensory pit, a field of sensilla simple without bordered margin and clypeus setose. Antennal segments are often very long and dark (rarely brightened) with coarse setae shorter than the segment diameter; the fourth flagellomere is often three to five times as long as it is wide (in some species, it is shorter—1.2–2.0 times). The flagellomere neck passes into the body without a distinct margin; therefore, flagellomeres appear flask-shaped. Thorax with coarse, strong and long setosity, and thoracic sclerites are not fused. The postpronotum is usually setose (sometimes bare), mesonotum has strong, long and coarse setae, scutellum, in addition to the numerous short setae, has four to seven very long marginal setae, and a high triangular catepisternum. The wing has macrotrichia on all branches of M and CuA. The membrane is bare or has some macrotrichia, especially at the apical part. R1 long, meeting C more or less opposite of M-fork; M-fork long, with narrow parallel, bent M1 and M2; stM shorter or almost the same length as M-fork; x longer than y; stCuA often indistinct, but if it is present, it is shorter than x. The anal lobe is well developed. Halter with a short stalk and large areas of multiple rows of bristles. Legs strong and long. The tibial organ of p1 has a triangular area of dense setae, not separated from the rest of the vestiture by a bare area or arcuate margin at the apex. The fore tibia is without spines among setae (exclude *S. hemerobioides*). The tibiae of p2 and p3 are two strong, slender and with equal in length spurs. The tarsal claws are strong, without teeth. Hypopygium with strong, compact and short gonocoxites. The intergonocoxal area has no central differentiation or bristle group, it only contains short setae. Gonostyles have a distinctly narrow base and are, therefore, club-shaped. The outer side of the apex of gonostyles usually has a coarse group of upward pointing spines; the inner side has no spines and is flat, without emargination (if gonostyles have emargination, then this is on the apex and gonostyles triangular); and the inner angle of the apex often has dense bristles on the subapical dark lobe. The apical tooth is missing, rarely gonostyles have a small beak-like tooth or large tooth-like lobe structure with several coarse spines. The tegmen is small, heavily sclerotized, distinctly higher than wide and has a high and rounded apex [4,7].

*Sciara* species are very similar to *Trichosia* Winnertz, 1867 and differences between the two genera are not always clear. The main distinguishing feature of *Sciara* is the flagellomere necks with seamless connection to the base and no clear margin between the neck and body; therefore, flagellomeres appear flask-shaped. *Trichosia* species have acute necks with a more or less clear margin [7].

### 3.2. Review of Collected Species

*Sciara analis* Schiner, 1864

Material examined. Ukraine, Ivano-Frankivsk region, outskirts of Bystrytsia, Forestry Office of Gorgany Nature Reserve: 48.49014° N, 24.27940° E, ca. 705 m a.s.l., anthropogenic meadowy cereals lawn, near tree trunk, with aspirator, 4 August 2020, two ♂, A. Babytskiy leg. (No. 3573-4).

Distribution. Albania, Austria, Czech Republic, France, Germany, Italy, Latvia, Lithuania, the Netherlands, Poland, Romania, the Russian Federation (European part), Sweden, Switzerland and Ukraine [2,8,14,15,18,32,33].

Chorology in Ukraine. Crimea, Lviv and Ivano-Frankivsk regions.

**Figure 1:**
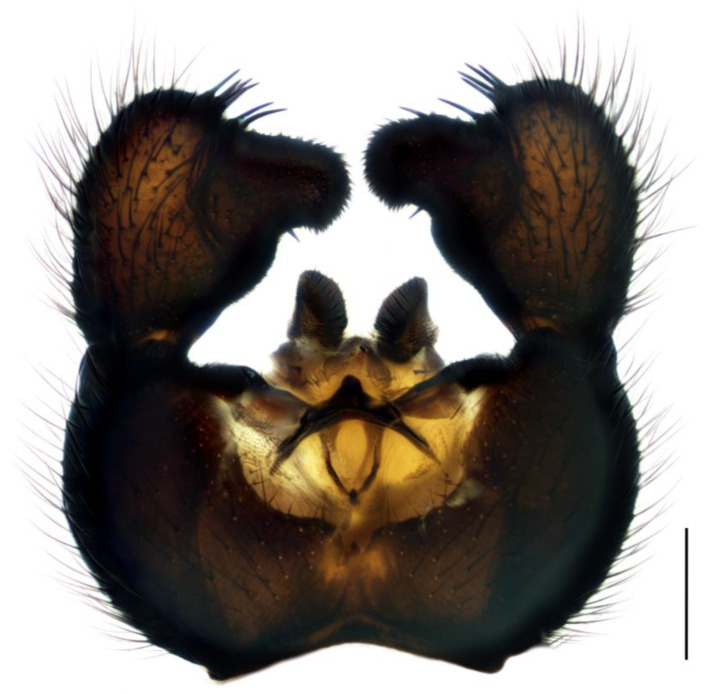
Hypopygium of *Sciara analis*, ventral view. Scale bar = 0.2 mm.

Diagnosis. Male adults reach four–six mm in length. Head black. Eye bridge in the middle is slightly separated. Dark-brown maxillary palpus and antennae. Flagellomeres relatively short, with dense setae, shorter than flagellomeres width and very short, weakly defined necks. Length/width of fourth flagellomere body = 1.95–2.26. Thorax black. Basal palpal segment elongated, about three/five times as long as the terminal segment, with numerous bristles, two–three times longer than others. Middle segment about as long as basal, with long bristles. Terminal segment much longer than other segments, with thin bristles. Legs dark brown, fore femur a little lighter. Length of spur/width of tibia: p1 = 1.23–1.55, p2 = 1.86–2.03 and p3 = 1.86–1.95. Length of metatarsus/length of tibia: p1 = 0.54–0.58, p2 = 0.48–0.50 and p3 = 0.44. Wing 4.2–4.6 mm long and 1.8–1.9 mm wide; width/length of wing = 0.40–0.45. Wing membrane dark, with sparse macrotrichia on the margin cells. R1 long, falls into C clearly apicad the base of M-fork, R1/R = 1.36–1.48; x bare, longer than y, x/y = 1.42–1.45, y with one macrotrichia near Rs; stM shorter than M-fork, with one–four macrotrichia, stM/M-fork = 0.79–0.89, M1 and M2 with numerous macrotrichia; CuA1 near the base becomes almost diaphanous by the half length of x, therefore, stCuA indistinct, CuA1 and CuA2 with numerous macrotrichia; c/w = 0.58–0.60. Halter dark brown. Hypopygium (Figure 1) black–brown. Gonocoxite wider than high, shorter than gonostylus, with long and dense bristles, inner side with short setae, membrane light-brown, bare only with a few short setae on the margin. Gonostylus club-shaped with a thin base and thick, rounded apex; inner angle of apex with large bulbous lobe covered with short dense bristles. The distance from the apical base of the lobe to its tip exceeds lobe tip thickness at most two times. Group of four–five upward pointed spines is in the inner apical side of gonostylus, one–two inward spines located lower, on the apical base of the lobe and sometimes one more spine is in the lobe middle, baso-medial lobe side with one–two downward spines.

Ecology. In Ukraine, it is known only for trampled habitat near fir and spruce forests of the lowland part of the forest belt on rich soils in the Carpathians. No habitats were given for the other published records.

Phenology. August.

*Sciara flavimana* Zetterstedt, 1851

Common synonyms. *S. fulgens* Winnertz, 1867; *S. mannii* Winnertz, 1867.

Material examined. Ukraine, Ternopil region, northern outskirts of Zhnyborody: 48.89407° N, 25.44164° E, ca. 270 m a.s.l., coastal vegetation on the spring bank, sweeping, 20 July 2019, one ♂, A. Babytskiy leg. (No. 1505); Ukraine, Volyn region, outskirts of Kholonevychi, Tsumanska Puscha National Nature Park: 51.00889° N, 25.92952° E, ca. 170 m a.s.l., wet meadow, sweeping, 13 August 2020, three ♂, A. Babytskiy leg. (No. 3763-5); Ukraine, Rivne region, eastern outskirts of Horodets: 51.27655° N, 26.36690° E, ca. 160 m a.s.l., wet meadow, sweeping, 10 August 2021, one ♂, A. Babytskiy leg. (No. 4075); Ukraine, Ternopil region, to south of Beremiany, Dniester Canyon National Nature Park, valley of Dniester River: 48.87431° N, 25.43947° E, ca. 320 m a.s.l., ecotone shrubs overgrowth on the edge of a hill, near field road and meadow, sweeping, 26 June 2021, one ♂, A. Babytskiy leg. (No. 4415); Ukraine, Ternopil region, between Kasperivtsi and Holihrady, left bank of Seret River: 48.68757° N, 25.87442° E, ca. 240 m a.s.l., pine and shrubs overgrowth, slope of hill, sweeping, 27 June 2021, two ♂, A. Babytskiy leg. (No. 4438, 4464); Ukraine, Ternopil region, to south of Beremiany, Chervona Hill, left bank of Seret River: 48.87431° N, 25.43947° E, ca. 320 m a.s.l., broad-leaved ribbon shrubs overgrowth, top of hill, sweeping, 28 June 2021, one ♂, A. Babytskiy leg. (No. 4479); Ukraine, Volyn region, outskirts of Solovychi: 51.06278° N, 24.48196° E, ca. 190 m a.s.l., artificial pine planting on the meliorated meadow, sweeping, 25 June 2022, one ♂, A. Babytskiy leg. (No. 5256).

Distribution. Austria, Croatia, Czech Republic, Denmark, Estonia, Finland, France (mainland, Corsica), Germany, Greece (mainland), Italy (mainland), Latvia, Norway, Poland, Romania, the Russian Federation (European part, Western Siberia (Altai)), Spain (mainland), Sweden, Switzerland, Turkmenistan (Central Kopet Dag), Ukraine (first record) and the United Kingdom [5,8,32,34,35].

Chorology in Ukraine. Ternopil, Rivne and Volyn regions.

**Figure 2:**
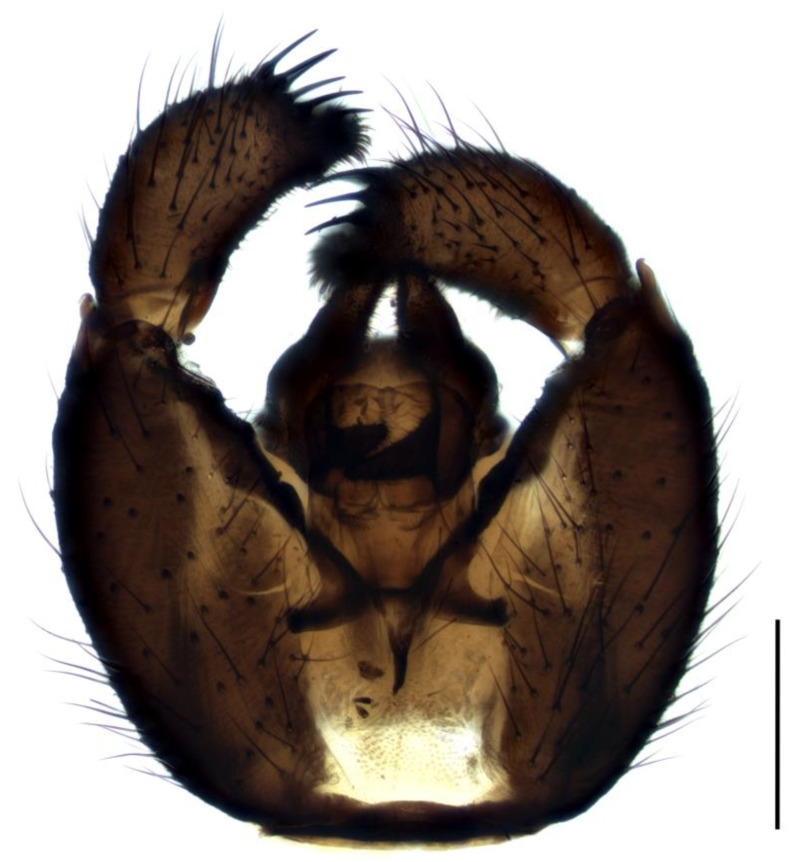
Hypopygium of *Sciara flavimana*, ventral view. Scale bar = 0.2 mm.

Diagnosis. Male adults reach 3.5–4.0 mm in length. Head dark-brown. Eye bridge wide and consists of four facet rows. Maxillary palpus and antennae brown. Flagellomeres relatively long, with dense setae, about as long as the flagellomeres width, and very short, weakly defined necks. Length/width of fourth flagellomere body = 3.09–3.42. Basal palpal segment with three–five long bristles. Middle segment as long as basal or a little shorter than basal, with bristles that are shorter than bristles on the basal segment. Terminal segment as long as basal or only up to 1.2 times as long as basal, with thin bristles. Thorax, abdomen and coxae dark brown, legs brown, fore femur and fore tibia lighter. Length of spur/width of tibia: p1 = 1.22–1.40, p2 = 1.63–2.18 and p3 = 1.56–2.01. Length of metatarsus/length of tibia: p1 = 0.53–0.58, p2 = 0.46–0.55 and p3 = 0.44–0.52. Wing 2.8–3.1 mm long (up to 4.0 [4]) and 1.0–1.2 mm wide, width/length of wing = 0.37–0.44. Wing membrane smoky-brown, with a few macrotrichia at the apex. R1 long, falls into C in front of the base of M-fork, R1/R = 1.12–1.32; x and y bare, x longer than y, x/y = 1.34–1.65; stM shorter than M-fork, weakly recognizable, with several macrotrichia at basal part, stM/M-fork = 0.84–0.90; M1 and M2 with numerous macrotrichia; in some specimens, CuA1 near the very base becomes almost diaphanous, but stCuA recognizable, stCuA/x = 0.50; CuA1 and CuA2 basally bare, from the half with macrotrichia; c/w = 0.72–0.74. Halter brown. Hypopygium (Figure 2) dark-brown. Gonocoxite higher than wide, distinctly longer than gonostylus, with wide intergonocoxal bare light-brown membrane and long setae. Gonostylus elongated, bean-shaped curved, with apical lobe, app two times narrower than the width of gonostylar apex. Gonostylus with a group of five–six spines located apically, above the base of lobe; lobe rounded, slightly elongate, covered with short dense bristles.

Ecology. In Ukraine, it is registered mostly on the meadow habitats or ecotone of grasslands and shrub or tree stands. All findings regard the following habitat types: herbaceous nitrophilous fringes of lowland rivers; wet eutrophic and mesotrophic hay grasslands; low- and medium-altitude hay meadows; ecotone of mesophilous and xeromesophilous shrubs and meadow steppes on calcareous soils (rendzina); and ecotone of cultivated area with coniferous and broad-leaved trees and anthropogenic transformed low- and medium-altitude hay meadows.

Phenology: June–August.

*Sciara hebes* (Loew, 1869)

Common synonyms. *S. marginata* Mohrig and Krivosheina, 1983 (preocc.); *S. mendax* Tuomikoski, 1960; *S. modesta* (Winnertz, 1867) (preocc.); *S. nursei* Freeman, 1983; and *S. ulrichi* Menzel and Mohrig, 1998.

Material examined. Ukraine, Ternopil region, outskirts of Krovinka: 49.33741° N, 25.67169° E, ca. 350 m a.s.l., hornbeam–oak–pine forest, quarry-like crater left after the explosion of the Wehrmacht ammunition depot, sweeping, 19 June 2019, one ♂, A. Babytskiy leg. (No. 4790).

Distribution. Austria, Belgium, Canada (Alberta, British Columbia, Ontario, Edmonton, Thebacha, Georgian Bay Islands, Gulf Islands, Inuvik, Manitoba, Norman Wells, Quebec, Saskatchewan and Yukon), China (Shaanxi), Czech Republic, Estonia, Finland, France, Ireland, Italy, the Netherlands, Norway, Poland, the Russian Federation (Khabarovsk Krai, Western Siberia (Altai)), Slovakia, Sweden, Ukraine (first record), the United Kingdom and USA (Michigan, Montana and New York) [5,8,27,29,32,36].

Chorology in Ukraine. Ternopil region.

**Figure 3:**
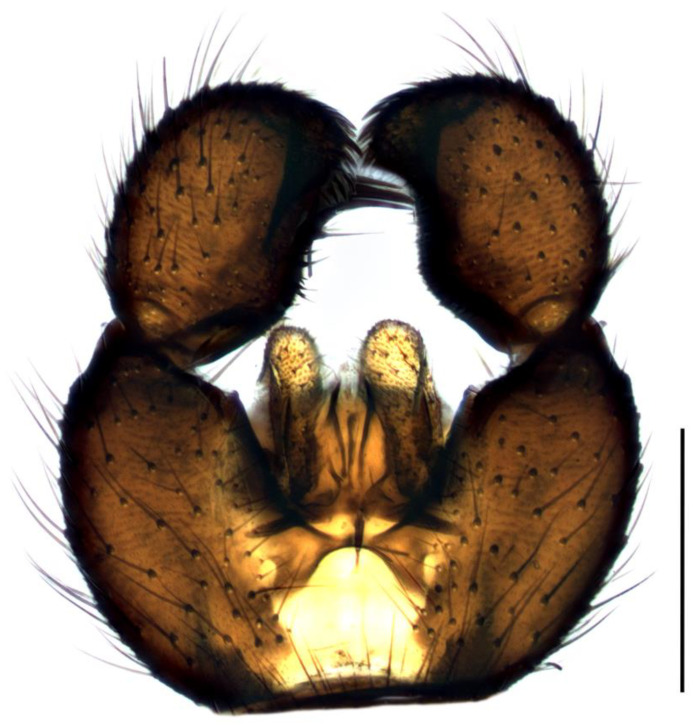
Hypopygium of *Sciara hebes*, ventral view. Scale bar = 0.2 mm.

Diagnosis. Male adults reach 3.0–3.5 mm in length. Head brown. Eye bridge consists of two–there facet rows. Maxillary palpus and antennae light-brown. Flagellomeres with dense setae, as long as the flagellomeres width or a little longer, necks clearly defined and slightly separated, at least 0.13 of flagellomere’s length. Length/width of fourth flagellomere body = 2.71–2.86. Basal palpal segment with five–six bristles. Middle segment oval-elongated, shorter than basal, with more than 10 short bristles and one longer. Terminal segment thinner and longer than basal, with numerous bristles. Thorax brown with yellow membranous areas on mesonotum. Coxae and legs yellow with darker tarsi and hind tibia. Length of spur/width of tibia: p1 = 1.48–1.69, p2 = 2.13–2.43 and p3 = 1.98–2.09. Length of metatarsus/length of tibia: p1 = 0.54–0.55, p2 = 0.46 and p3 = 0.44–0.46. Wing 2.5 mm long (3.0–3.5 mm [4]) and 1.0 mm wide, width/length of wing = 0.40. Wing membrane slightly smoky, with numerous macrotrichia at the apical half. R1 long, falls into C in front of the base of M-fork, R1/R = 1.40; x bare, shorter than y, y with one macrotrichia near the base of stM or bare, x/y = 0.97; stM shorter than M-fork, weakly recognizable, with about 10 macrotrichia, stM/M-fork = 0.84; M1 and M2 with numerous macrotrichia; stCuA short, stCuA/x = 0.61; CuA1 and CuA2 with macrotrichia; c/w = 0.67. Halter brown. Hypopygium (Figure 3) brown. Gonocoxite wider than high, as long as gonostylus, with long bristles and deep intergonocoxal bare light-brown membrane. Gonostylus bean-shaped, bulbous at the base, extended ventrally to the tip to form a broadly rounded lobe, covered with short dense bristles. On the dorsal side of gonostylus, inside at the base of the ventral lobe, is a clearly projecting hump with a group of three–six long black spines (one of them sometimes slightly shorter and more isolated standing at the upper base of the hump [27]); one or two more shorter spines are ventrally, on the apex of lobe.

Ecology. In Ukraine, it is known only for Central European oak–hornbeam forests.

Phenology. June.

*Sciara helvola* Winnertz, 1867

Material examined. Ukraine, Cherkasy region, outskirts of Kaniv, Kaniv Natural Reserve: 49.72734° N, 31.51852° E, ca. 200 m a.s.l., oak–hornbeam forest, sweeping, 20 June 2015, one ♂, A. Babytskiy leg. (No. 131); Ukraine, Cherkasy region, outskirts of Kaniv, Kaniv Natural Reserve: 49.72804° N, 31.51455° E, ca. 200 m a.s.l., ecotone steppe lawn in a broad-leaved forest, sweeping on trees, 5 June 2019, one ♂, A. Babytskiy leg. (No. 2335); Ukraine, Cherkasy region, outskirts of Kaniv, Kaniv Natural Reserve: 49.71939° N, 31.50180° E, ca. 220 m a.s.l., oak–hornbeam forest, sweeping, 6 June 2019, seven ♂, A. Babytskiy leg. (No. 2418, 2435, 2445, 2490-1, 2516, 2525); Ukraine, Cherkasy region, outskirts of Kaniv, Kaniv Natural Reserve: 49.72265° N, 31.53011° E, ca. 200 m a.s.l., maple–hornbeam forest, sweeping, 7 June 2019, one ♂, A. Babytskiy leg. (No. 2983); Ukraine, Vinnytsia region, southern outskirts of Frankivka, Dniester River valley: 50.57548° N, 27.61996° E, ca. 80 m a.s.l., bottom of ravine with spring, overgrown by trees and shrubs, sweeping, 19 June 2021, 31 ♂, A. Babytskiy leg. (No. 4080-7, 4089-96, 4098-106, 4108-13); Ukraine, Vinnytsia region, Busha: 48.34025° N, 28.11142° E, ca. 145 m a.s.l., steppe slope of a hill, near wood along a forest road, sweeping, 20 June 2021, one ♂, A. Babytskiy leg. (No. 4123); Ukraine, Vinnytsia region, northern outskirts of Kozliv: 48.48867° N, 27.52140° E, ca. 180 m a.s.l., roadside wood plantation, sweeping, 21 June 2021, one ♂, A. Babytskiy leg. (No. 4136); Ukraine, Chernivtsi region, northern outskirts of Novodnistrovsk, bank of Dniester River, Khotyn National Nature Park: 48.58870° N, 27.45185° E, ca. 150 m a.s.l., oak–hornbeam forest, sweeping, 21 June 2021, 51 ♂, A. Babytskiy leg. (No. 4145, 4148, 4151-3, 4158, 4161, 4166-7, 4171, 4174, 4189-90, 4196-8, 4200, 4202, 4204, 4206, 4208-9, 4213-6, 4220-4, 4226-7, 4230, 4232, 4235, 4237, 4240, 4242-4, 4247-54, 4257, 4259); Ukraine, Chernivtsi region, northeastern outskirts of Spaska: 48.29888° N, 25.77772° E, ca. 440 m a.s.l., tall-grass wet meadow, forested ravine, sweeping, 22 June 2021, 15 ♂, A. Babytskiy leg. (No. 4266-7, 4272-3, 4278-80, 4282, 4286-8, 4291, 4293-4, 4296); Ukraine, Ternopil region, southern outskirts of Babyntsi, Dniester Canyon National Nature Park, valley of Dniester River: 48.65057° N, 26.04671° E, ca. 220 m a.s.l., oak–hornbeam forest, slope of hill, near the source of spring, sweeping, 25 June 2021, two ♂, A. Babytskiy leg. (No. 4396, 4399); Ukraine, Ternopil region, between Kasperivtsi and Holihrady, left bank of Seret River: 48.68757° N, 25.87442° E, ca. 240 m a.s.l., pine and shrubs overgrowth, slope of hill, sweeping, 27 June 2021, five ♂, A. Babytskiy leg. (No. 4429, 4436, 4451, 4457, 4469); Ukraine, Ternopil region, to south of Beremiany, Chervona Hill, left bank of Seret River: 48.87431° N, 25.43947° E, ca. 320 m a.s.l., broad-leaved ribbon shrubs overgrowth, top of hill, sweeping, 28 June 2021, one ♂, A. Babytskiy leg. (No. 4501); Volyn region, between Zhuravychi and Klubochyn, Tsumanska Puscha National Nature Park: 50.96952° N, 25.75357° E, ca. 200 m a.s.l., hornbeam–birch forest with oak, along the forest road, sweeping, 28 June 2022, 10 ♂, A. Babytskiy leg. (No. 5333-4, 5351, 5359, 5364, 5372, 5392, 5412, 5422-3).

Distribution. Bulgaria, Czech Republic, Finland, Germany, Japan (Kanagawa), Latvia, Norway, Poland, the Russian Federation (European part, western Siberia (Altai)), Slovakia, South Korea, Sweden, Switzerland and Ukraine (first record) [8,32,35,37].

Chorology in Ukraine. Cherkasy, Chernivtsi, Ternopil, Vinnytsia and Volyn regions.

**Figure 4:**
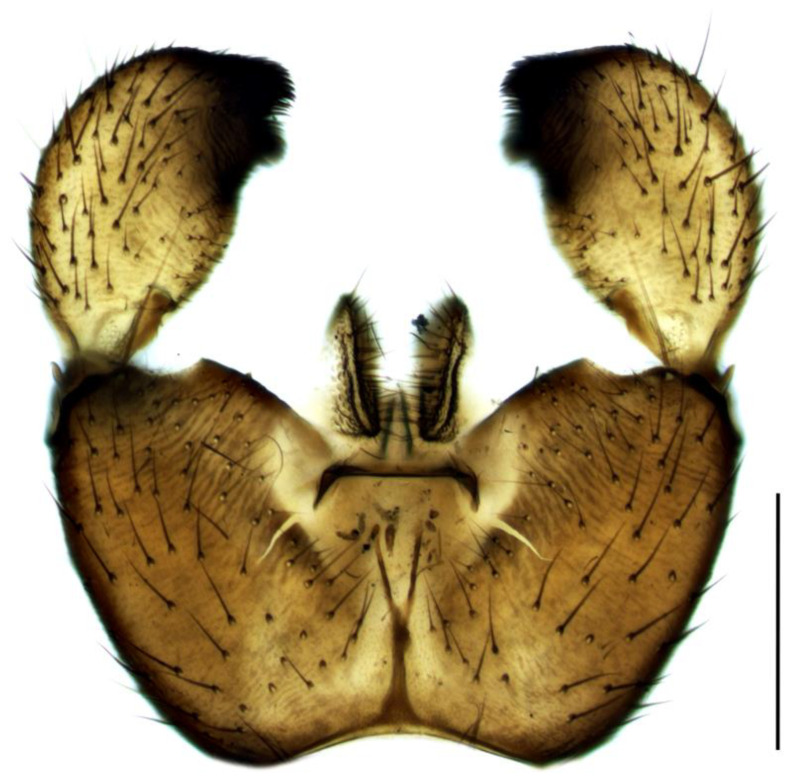
Hypopygium of *Sciara helvola*, ventral view. Scale bar = 0.2 mm.

Diagnosis. Male adults reach 3.5–4.8 mm in length. Head brown. Eye bridge consists of two–three facet rows. Antennae light-brown with yellow scape, pedicel and the base of the first flagellomere. Flagellomeres with dense light setae, as long as the flagellomeres width, and very short, weakly defined necks. Length/width of fourth flagellomere body = 3.18–3.72. Basal palpal segment brown, with five–eight bristles. Middle segment brown, shorter than basal, with bristles shorter than bristles on the basal segment. Terminal segment light-brown, clearly longer than basal, with app 10 thin bristles. Thorax dark-brown with lighter membranous areas on mesonotum. Coxae light-brown, legs yellow with darker tarsi. Length of spur/width of tibia: p1 = 1.23–1.45, p2 = 1.63–1.92 and p3 = 1.52–1.69. Length of metatarsus/length of tibia: p1 = 0.48–0.54, p2 = 0.41–0.48 and p3 = 0.40–0.45. Wing 2.3–3.1 mm long (4.5 mm [26]) and 0.9–1.1 mm wide, width/length of wing = 0.33–0.39. Wing membrane slightly smoky, without macrotrichia. R1 falls into C before or in front of the base of M-fork, R1/R = 1.10–1.39; x shorter than y, both bare, x/y = 0.85–0.96; stM indistinct, without macrotrichia, about as long as M-fork, stM/M-fork = 0.90–1.08; M1 and M2 with numerous macrotrichia; stCuA indistinct or very short, makes at most 0.3 x; CuA1 and CuA2 in basal part bare, with distal macrotrichia; c/w = 0.72–0.80. Halter brown. Hypopygium (Figure 4) light-brown to yellow, with darker gonostylar lobe. Gonocoxite higher than wide, longer than gonostylus, with short bristles and a triangular bare yellow intergonocoxal membrane; upper angle of gonocoxite with one long bristle (longer than a half of gonostylus). Gonostylus oval, with a thin base and slightly curved inward apex with wide dark subapical lobe covered with short dense bristles. Lobe with wide concave tip margin and, on some specimens, appears double or duplex.

Ecology. In Ukraine, it is registered mostly in forests or ecotones of grasslands and shrub or tree stands, but with clear gravitation to dendrobium. All findings regard the following habitat types: Central European oak–hornbeam forests; anthropogenic broad-leaved forests; mesophilous fringes on acidic soils; ecotone of mesophilous and xeromesophilous shrubs; and meadow steppes on calcareous soils (rendzina).

Phenology. June.

*Sciara hemerobioides* (Scopoli, 1763)

Common synonyms. *S. lateralis* Meigen, 1818; *S. morio* (Fabricius, 1794); *S. thomae* (Linnaeus, 1767); and *S. valida* Winnertz, 1867.

Material examined. Ukraine, Ivano-Frankivsk region, northern outskirts of Nezvysko, the left bank of Dniester River: 48.78303° N, 25.25203° E, ca. 200 m a.s.l., coastal beech forest, sweeping around the spring sources, 10 August 2016, one ♂, A. Babytskiy leg. (No. 56); Ukraine, Ternopil region, northeast outskirts of Nyrkiv: 48.82221° N, 25.57476° E, ca. 230 m a.s.l., oak–hornbeam forest, along the forest road, sweeping, 22 July 2019, one ♂, A. Babytskiy leg. (No. 1585); Ukraine, Zakarpatska region, outskirts of Mala Uholka, Carpathian Biosphere Reserve: 48.25609° N, 23.62223° E, ca. 475 m a.s.l., beech primeval forest, aged plantation of *Pseudotsuga* Carr., sweeping, 2 August 2019, one ♂, A. Babytskiy leg. (No. 1734); Ukraine, Zakarpatska region, outskirts of Mala Uholka, Carpathian Biosphere Reserve: 48.26364° N, 23.61694° E, ca. 690 m a.s.l., beech primeval forest, sweeping, 2 August 2019, 11 ♂, A. Babytskiy leg. (No. 1735-41, 1743, 1749-50); Ukraine, Zakarpatska region, outskirts of Mala Uholka, Carpathian Biosphere Reserve: 48.26694° N, 23.62933° E, ca. 610 m a.s.l., beech primeval forest, rocky outcrops, sweeping, 3 August 2019, one ♂, A. Babytskiy leg. (No. 1798); Ukraine, Zakarpatska region, outskirts of Mala Uholka, Carpathian Biosphere Reserve: 48.26625° N, 23.62984° E, ca. 550 m a.s.l., beech primeval forest, sweeping, 3 August 2019, eight ♂, A. Babytskiy leg. (No. 1810, 1817, 1819, 1837-8, 1843, 1846-7); Ukraine, Zakarpatska region, outskirts of Mala Uholka, Carpathian Biosphere Reserve: 48.26942° N, 23.63465° E, ca. 540 m a.s.l., beech primeval forest, undisturbed channel wood, sweeping, 4 August 2019, two ♂, A. Babytskiy leg. (No. 1894-5); Ukraine, Zakarpatska region, outskirts of Mala Uholka, Carpathian Biosphere Reserve: 48.26954° N, 23.63552° E, ca. 530 m a.s.l., beech primeval forest, anthropogenically load channel wood, sweeping, 4 August 2019, one ♂, A. Babytskiy leg. (No. 1941); Ukraine, Zakarpatska region, outskirts of Mala Uholka, Carpathian Biosphere Reserve: 48.27708° N, 23.64047° E, ca. 600 m a.s.l., beech primeval forest, sweeping, 4 August 2019, one ♂, A. Babytskiy leg. (No. 1963); Ukraine, Zakarpatska region, outskirts of Velyka Uholka, Carpathian Biosphere Reserve: 48.25619° N, 23.67759° E, ca. 840 m a.s.l., beech primeval forest, near cave, sweeping, 5 August 2019, three ♂, A. Babytskiy leg. (No. 2033, 2100-1); Ukraine, Zakarpatska region, outskirts of Mala Uholka, Carpathian Biosphere Reserve: 48.2665° N, 23.62984° E, ca. 550 m a.s.l., beech primeval forest, from clothes of man, during the copulation process, with aspirator, 6 August 2019, one ♂, one ♀, A. Babytskiy leg. (No. 2108); Ukraine, Ternopil region, Zaliztsi, bank of the lake on Seret River: 49.79788° N, 25.37057° E, ca. 320 m a.s.l., coastal ruderal shrubs on a dam, tall grasses, sweeping, 1 August 2022, one ♂, A. Babytskiy leg. (No. 5524).

Distribution. Austria, Belarus, Belgium, Bosnia and Herzegovina, Czech Republic, Denmark, Estonia, Finland, France (mainland, Corsica), Germany, Indonesia (Sumatra), Ireland, Italy (mainland), Japan (Kanagawa), Latvia, Lithuania, the Netherlands, Norway, Poland, Romania, the Russian Federation (European part, eastern and western Siberia (northern Altai)), Slovenia, Slovakia, Spain (mainland, Canary Is.), Sweden, Switzerland, Taiwan, Ukraine and the United Kingdom [2,5,8,13,14,15,18,19,32,35,38].

Chorology in Ukraine. Crimea, Lviv, Ivano-Frankivsk, Ternopil and Zakarpatska regions.

**Figure 5:**
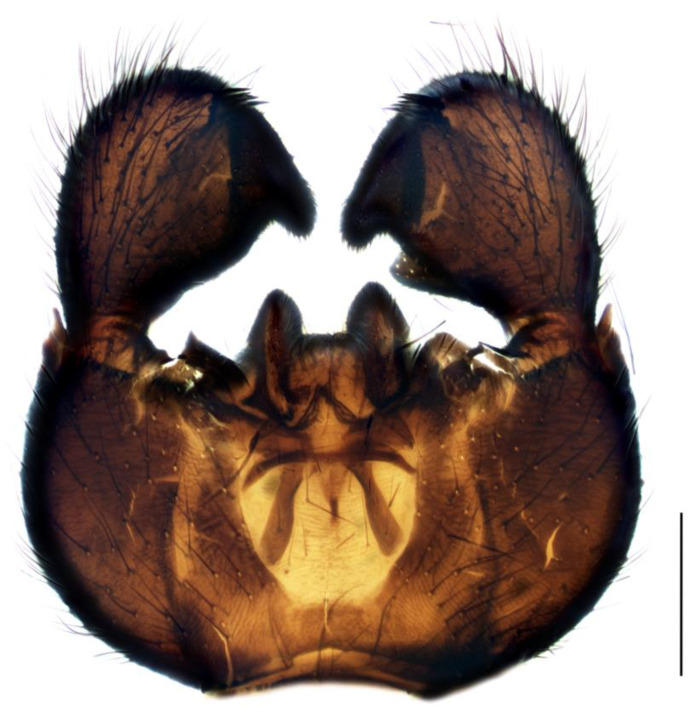
Hypopygium of *Sciara hemerobioides*, ventral view. Scale bar = 0.2 mm.

Diagnosis. Male adults reach four–five mm in length. Head black. Eye bridge wide, consists of four facet rows. Maxillary palpus and antennae dark brown. Flagellomeres relatively long, with dense setae, about as long as the flagellomeres width, and very short, weakly defined necks. Length/width of fourth flagellomere body = 2.7–3.2 (in Ukrainian specimens, 3.01–3.32). Basal palpal segment narrow, about 4/5 times as long as the terminal, with several long bristles, one–three times longer than others. Middle segment shorter than basal, with long bristles. Terminal segment 1.6 times as long as middle segment, with thin bristles. Thorax, abdomen, coxae, and legs unicolored dark brown to black, fore femur a little lighter. Coxae short, legs very long and slender. Length of spur/width of tibia: p1 = 1.21–1.35, p2 = 1.83–2.03 and p3 = 1.70–2.11. Length of metatarsus/length of tibia: p1 = 0.48–0.55, p2 = 0.44–0.49 and p3 = 0.43–0.47. Wing 3.9–4.4 mm long and 1.5–1.7 mm wide, width/length of wing = 0.38–0.40. Wing membrane dark-brown , apically with macrotrichia. R1 long, falls into C in front of the base of M-fork, R1/R = 1.18–1.40; x bare, longer than y, x/y = 1.22–1.47, y bare or with one macrotrichia near Rs; stM shorter than M-fork, bare, stM/M-fork = 0.71–0.91; M1 and M2 with numerous macrotrichia; CuA1 near the base becomes almost diaphanous by the half length of x, therefore, stCuA indistinct, CuA1 and CuA2 with numerous macrotrichia; c/w = 0.55–0.60. Halter dark-brown. Hypopygium (Figure 5) black-brown. Gonocoxite wider than high, as long as gonostylus, inner side of gonocoxites evenly rounded, with very short setae, membrane light-brown, bare only with a few short marginal setae. Gonostylus club-shaped, with thin base and thick, rounded apex; outside of the gonostyles evenly rounded and apically with a group of five–eight spines and a tongue-shaped, triangular lobe covered with short dense bristles. The distance from the apical base of the lobe to its tip exceeds lobe tip thickness much more than two times.

Ecology. In Ukraine, it is recorded mostly in broad-leaved forests in the western part, but one registration was in anthropogenically transformed ruderal tall grasslands near the bank of a lake. Also, females are often found sitting on the tall vegetation of grasslands [13,39], but species identification of *S. hemerobioides* based on female examination is not reliable because this species can be easily confused with *S. analis*. Therefore, even considering the much less frequent occurrence of *S. analis* than *S. hemerobioides* in Ukraine, we did not add female registrations to the species spreading. All findings regard the following habitat types: Central European oak–hornbeam forests; anthropogenic coniferous forests with *Pseudotsuga*; Central European neutrophilous beech forests; and herbaceous nitrophilous fringes of lowland rivers.

Phenology. July–August.

*Sciara humeralis* Zetterstedt, 1851

Common synonyms. *S. analis* var. *bezzii* Del Guercio, 1905; *S. armata* Winnertz, 1867; and *S. hamatilis* Yang, Zhang and Yang, 1993.

Material examined. Ukraine, Volyn region, outskirts of Berestiane, Tsumanska Puscha National Nature Park: 50.97375° N, 25.94138° E, ca. 180 m a.s.l., wet haymaking meadow, sweeping, 12 August 2020, two ♂, A. Babytskiy leg. (No. 3694, 3697); Ukraine, Volyn region, outskirts of Berestiane, Tsumanska Puscha National Nature Park: 50.94063° N, 25.95123° E, ca. 180 m a.s.l., wet haymaking meadow, sweeping, 12 August 2020, one ♂, A. Babytskiy leg. (No. 3719); Ukraine, Volyn region, outskirts of Berestiane, Tsumanska Puscha National Nature Park: 50.94215° N, 25.95353° E, ca. 180 m a.s.l., wet haymaking meadow, sweeping, 12 August 2020, one ♂, A. Babytskiy leg. (No. 3733); Ukraine, Volyn region, outskirts of Berestiane, Tsumanska Puscha National Nature Park: 50.97194° N, 25.94257° E, ca. 180 m a.s.l., coastal meadow, sweeping, 13 August 2020, three ♂, A. Babytskiy leg. (No. 3782-4); Ukraine, Volyn region, outskirts of Vorotniv, Vorotniv Botanical Reserve: 50.72240° N, 25.56622° E, ca. 230 m a.s.l., meadow, dam between ponds, sweeping, 16 August 2019, three ♂, A. Babytskiy leg. (No. 3865, 3870, 3876); Ukraine, Volyn region, southern outskirts of Nevir, Prypiat River valley: 51.86719° N, 24.98927° E, ca. 220 m a.s.l., wet meadow, sweeping, 7 August 2021, five ♂, A. Babytskiy leg. (No. 4040-4).

Distribution. Albania, Austria, Bulgaria, Belgium, Canada (Manitoba, New Brunswick, Newfoundland and Labrador, Nova Scotia, Ontario, Prince Edward Island and Quebec), Czech Republic, Denmark, Estonia, Finland, France, Germany, Hungary, Ireland, Italy (mainland), Japan (Kagoshima), Latvia, Lithuania, the Netherlands, Norway, Poland, Romania, the Russian Federation (European part, west Siberia (Altai)), Slovakia, South Korea, Sweden, Taiwan, Ukraine, the United Kingdom and USA (New York and Oklahoma) [2,5,8,18,32,35,40].

Chorology in Ukraine. Crimea and Volyn regions.

**Figure 6:**
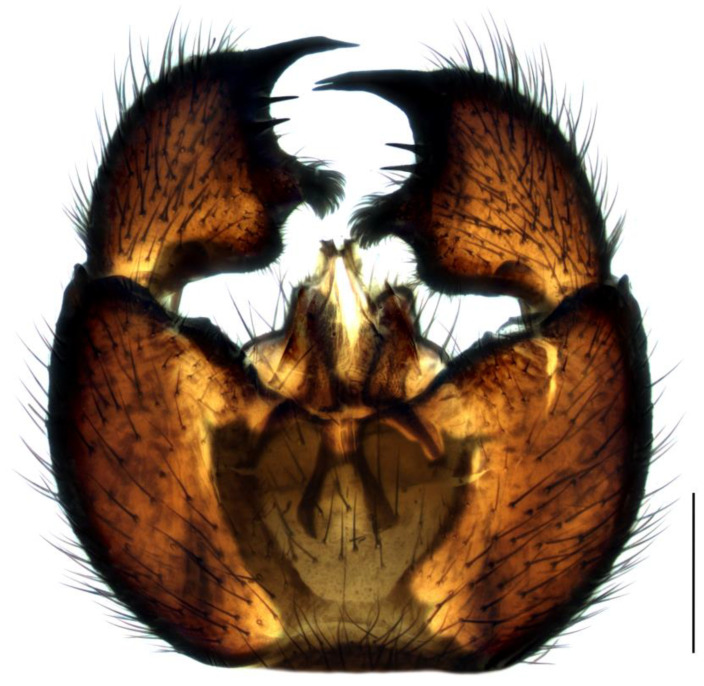
Hypopygium of *Sciara humeralis*, ventral view. Scale bar = 0.2 mm.

Diagnosis. Male adults reach 3.3–4.4 mm in length [7]. Head black . Eye bridge consists of two–three facet rows. Maxillary palpus and antennae dark-brown . Flagellomeres relatively long, with dense setae, about as long as the flagellomeres width, and short, weakly defined necks. Length/width of fourth flagellomere body = 2.55–3.27. Basal palpal segment with several long bristles. Middle segment shorter than others, with four–five long and several short bristles. Terminal segment as long as basal, with thin bristles. Thorax reddish-brown to black–brown, often with lateral brightening; abdomen reddish- to dark-brown. Coxae brown, legs yellow with darker tarsi, metatarsus yellow with brown apical part. Length of spur/width of tibia: p1 = 1.19–1.31, p2 = 1.87–2.01 and p3 = 1.59–1.81. Length of metatarsus/length of tibia: p1 = 0.50–0.56, p2 = 0.43–0.47 and p3 = 0.43–0.45. Wing 3.0–3.3 mm long and 1.1–1.3 mm wide, width/length of wing = 0.37–0.40. Wing membrane brown, without macrotrichia. R1 long, falls into C in front of the base of M-fork, R1/R = 1.15–1.22; x bare, longer than y, x/y = 1.38–1.60, y with two–three macrotrichia; stM about as long as M-fork with several macrotrichia in apical part, stM/M-fork = 0.89–1.01; M1 and M2 with numerous macrotrichia located very close to each other; stCuA indistinct, CuA1 and CuA2 with numerous macrotrichia located very close to each other; c/w = 0.67–0.71. Halter brown. Hypopygium (Figure 6) round, yellowish-brown. Gonocoxite higher than wide, longer than gonostylus, with short bristles and U-shaped bare yellow intergonocoxal membrane. Gonostylus compact, triangular, with coarse, long bristles on the outside, and two lobes on the inner side—apical large, tooth-like, saber-shaped lobe and small, tongue-shaped baso-medial lobe. Gonostylus deep excavated and here, with one to four strong spines on high sockets (position and number of spines often vary and can be different even on the left and right gonostyli). The base of the saber-shaped lobe is apically flat (gonostylus smoothly passes into the lobe), or with an apical notch whose depth varies in different specimens. The apical saber-shaped lobe wears two terminal spines (then, the lobe appears cloven), but in some specimens, there are three–four terminal spines [7]. Baso-medial, tongue-shaped lobe located in the lower part of the gonostylar inner side and densely covered with coarse and very dark bristles.

Ecology. In Ukraine, it is registered as meadowy species, known only from grasslands. All findings regard two habitat types: wet eutrophic and mesotrophic hay grasslands, and wet pastures.

Phenology. August.

*Sciara incerta* Winnertz, 1867

Common synonyms. *S. piriformis* Antonova, 1978 and *S. tibialis* Winnertz, 1867.

Material examined. Ukraine, Ternopil region, outskirts of Kasperivtsi, bank of Kasperivtsi Reservoir on Seret River: 48.67120° N, 25.85270° E, ca. 170 m a.s.l., coastal meadowy lawn surrounded by forest, sweeping, 22 June 2018, one ♂, A. Babytskiy leg. (No. 646); Ukraine, Ternopil region, outskirts of Kasperivtsi, bank of Kasperivtsi Reservoir on Seret River: 48.67120° N, 25.85270° E, ca. 170 m a.s.l., coastal meadowy lawn surrounded by forest, sweeping, 24 June 2018, one ♂, A. Babytskiy leg. (No. 667); Ukraine, Ternopil region, southern outskirts of Babyntsi, Dniester Canyon National Nature Park, near natural monument “Bilyi Kamin”, valley of Dniester River: 48.64989° N, 26.05343° E, ca. 250 m a.s.l., hay meadow steppe, slope of hill, sweeping, 25 June 2021, one ♂, A. Babytskiy leg. (No. 4345); Ukraine, Ternopil region, southern outskirts of Babyntsi, Dniester Canyon National Nature Park, near natural monument “Bilyi Kamin”, valley of Dniester River: 48.64970° N, 26.05253° E, ca. 230 m a.s.l., wood sinusia in meadow steppe, slope of hill, sweeping, 25 June 2021, two ♂, A. Babytskiy leg. (No. 4366, 4386); Ukraine, Ternopil region, to south of Beremiany, Dniester Canyon National Nature Park, valley of Dniester River: 48.87431° N, 25.43947° E, ca. 320 m a.s.l., ecotone shrubs overgrowth on the edge of a hill, near field road and meadow, sweeping, 26 June 2021, three ♂, A. Babytskiy leg. (No. 4414, 4418, 4421); Ukraine, Ternopil region, between Kasperivtsi and Holihrady, left bank of Seret River: 48.68757° N, 25.87442° E, ca. 240 m a.s.l., pine and shrubs overgrowth, slope of hill, sweeping, 27 June 2021, two ♂, A. Babytskiy leg. (No. 4452, 4461).

Distribution. Albania, Czech Republic, Germany, Spain (mainland), the Russian Federation (Krasnodar Krai) and Ukraine (first record) [2,41].

Chorology in Ukraine. Ternopil region.

**Figure 7:**
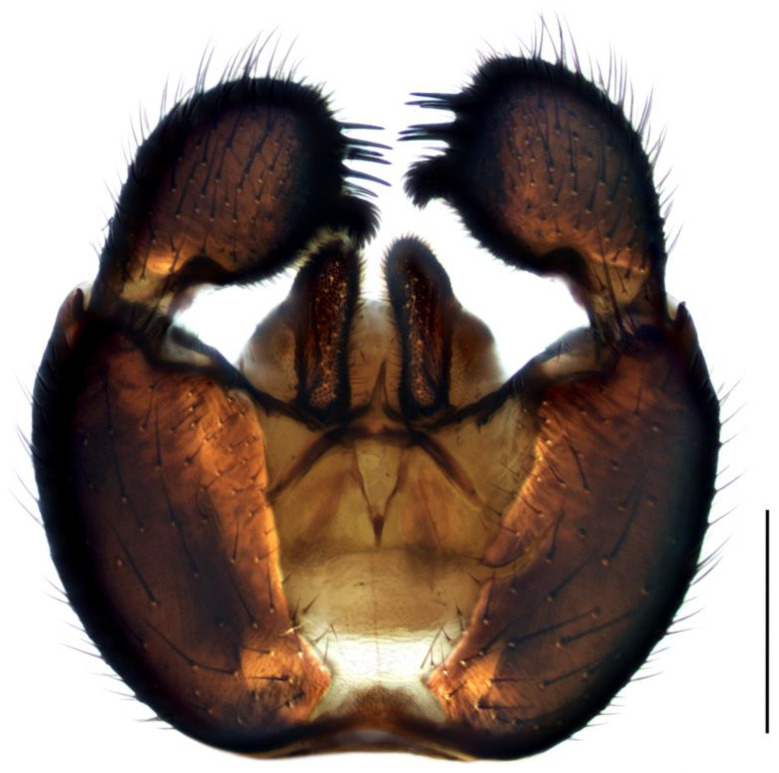
Hypopygium of *Sciara incerta*, ventral view. Scale bar = 0.2 mm.

Diagnosis. Male adults reach 3.0–3.5 mm in length. Head dark-brown. Eye bridge consists of four facet rows [2]. Maxillary palpus and antennae brown. Flagellomeres relatively long, with dense protruding setae, as long as the flagellomeres width or shorter; necks short, weakly defined. Length/width of fourth flagellomere body = 3.11–3.66. Basal palpal segment clubby, with three–six long bristles. Middle segment shorter than basal, with bristles, shorter than the bristles on the basal segment. Terminal segment clearly longer than basal, with three–four long and several short bristles. Thorax dark-brown. Coxae brown, legs yellow with darker tarsi. Length of spur/width of tibia: p1 = 1.18–1.25, p2 = 1.61–1.73 and p3 = 1.69–1.93. Length of metatarsus/length of tibia: p1 = 0.48–0.56, p2 = 0.43–0.50 and p3 = 0.44–0.47. Wing 2.6–2.9 mm long (3.3 mm [2]) and 1.0–1.1 mm wide, width/length of wing = 0.36–0.39. Wing membrane brown, without macrotrichia. R1 falls into C in front of the base of M-fork, R1/R = 1.29–1.44; x bare, longer than y, x/y = 1.52–1.63, y with three–six macrotrichia; stM indistinct, without macrotrichia, about as long as M-fork or little shorter, stM/M-fork = 0.92–1.03; M1 and M2 with sparse macrotrichia; CuA1 bare or with one–two macrotrichia, near the very base becomes almost diaphanous, therefore, stCuA is indistinct, CuA2 with sparse macrotrichia; c/w = 0.61–0.62. Halter brown. Hypopygium (Figure 7) round and brown. Gonocoxite higher than wide, longer than gonostylus, with long bristles, inner side with short setae; membrane yellow, bare, only with a few short setae on the margin. Gonostylus club-like, with a very thin base and thick, rounded apex with a group of five–six spines; under the group of spines, on the inner angle of the apex located a bulbous lobe covered with short dense bristles and several longer bristles on raised sockets above the lobe.

Ecology. In Ukraine, it is registered in meadowy and forestry habitats, also in the ecotone between grasslands and woods. All findings regard the following habitat types: Central European oak–hornbeam forests; meadow steppes on calcareous soils (rendzina); ecotone of mesophilous and xeromesophilous shrubs and meadow steppes on calcareous soils (rendzina); and ecotone between anthropogenic broad-leaved forests and herbaceous nitrophilous fringes of lowland rivers.

Phenology. June.

*Sciara ruficauda* Meigen, 1818

Common synonyms. *S. boleti* Winnertz, 1867; *S. mamaevi* Antonova, 1978; and *S. vigilax* Winnertz, 1867.

Material examined. Ukraine, Ivano-Frankivsk region, outskirts of Bystrytsia, Gorgany Nature Reserve: 48.46648° N, 24.32005° E, ca. 1430 m a.s.l., spruce forest, along a forest road, sweeping, 5 August 2020, one ♂, A. Babytskiy leg. (No. 3622); Ukraine, Volyn region, outskirts of Berestiane, Tsumanska Puscha National Nature Park: 50.97194° N, 25.94257° E, ca. 180 m a.s.l., coastal meadow, sweeping, 13 August 2020, one ♂, A. Babytskiy leg. (No. 3785); Ukraine, Volyn region, northern outskirts of Trostianets and Yaromel, Tsumanska Puscha National Nature Park: 50.96214° N, 25.59570° E, ca. 230 m a.s.l., pine forest with add of oak and birch, sweeping, 27 June 2022, one ♂, A. Babytskiy leg. (No. 5259).

Distribution. Austria, Czech Republic, Denmark, Finland, Germany, the Netherlands, Norway, Poland, the Russian Federation (Amur Krai, western Siberia (Altai)), South Korea, Spain (mainland), Sweden, Ukraine and the United Kingdom [2,5,8,13,18,32,35,42].

Chorology in Ukraine. Crimea, Ivano-Frankivsk, Kyiv and Volyn regions.

**Figure 8:**
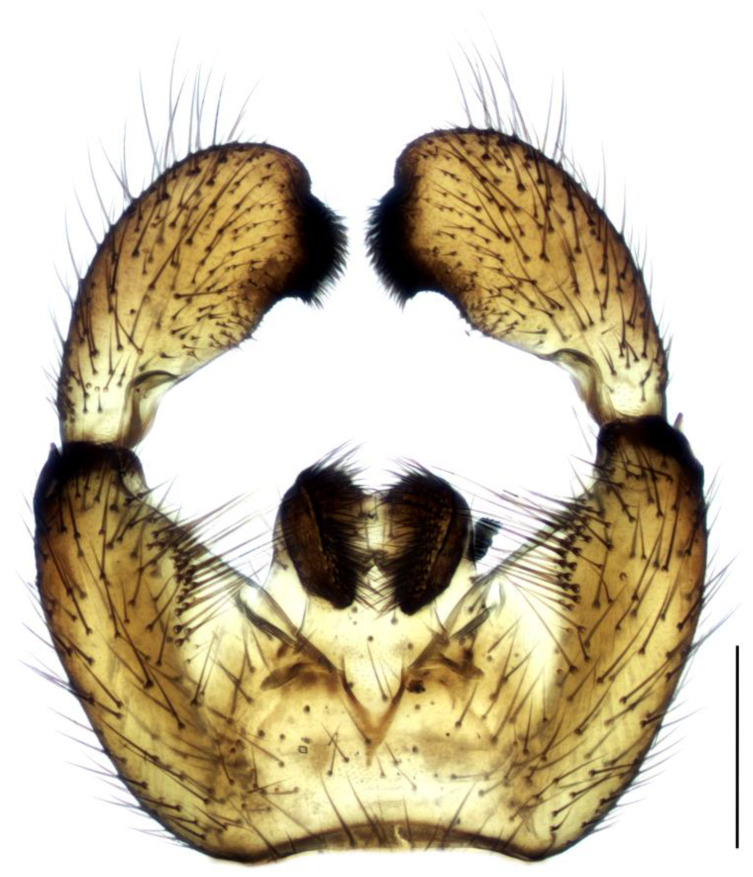
Hypopygium of *Sciara ruficauda*, ventral view. Scale bar = 0.2 mm.

Diagnosis. Male adults reach 3.5–4.5 mm in length. Head brown . Eye bridge wide, consists of three–four facet rows. Antennae light-brown with yellow scapus, pedicel and basal parts of first and second flagellomeres. Flagellomeres relatively long, with protruding bright setae, about as long as the flagellomeres width; necks short, weakly defined. Length/width of fourth flagellomere body = 3.57–4.13. Maxillary palpus light-brown, basal palpal elongated with several long bristles. Middle segment shorter than basal, with three–four long and several short bristles. Terminal segment much longer than basal, with several bristles. Thorax dark-brown with yellow membranous areas on mesonotum. Abdomen brown, slightly lighter than thorax. Coxae reddish-yellow, legs yellow with darker tarsi, metatarsus yellow with brown apical part. Length of spur/width of tibia: p1 = 1.23–1.37, p2 = 1.81–2.02 and p3 = 1.71–2.11. Length of metatarsus/length of tibia: p1 = 0.57–0.60, p2 = 0.46–0.48 and p3 = 0.43–0.47. Wing 2.8–3.1 mm long and 1.1–1.2 mm wide, width/length of wing = 0.37–0.39. Wing membrane smoky-brown, with a few sparse macrotrichia at the apex. R1 falls into C in front of the base of M-fork or slightly basad, R1/R = 1.13–1.37; x shorter than y, both bare, x/y = 0.88–0.92; stM about as long as M-fork or a little longer, very hard to recognize, with several macrotrichia, stM/M-fork = 1.00–1.05; M1 and M2 with numerous macrotrichia; stCuA indistinct; CuA1 and CuA2 with macrotrichia; c/w = 0.64–0.70. Halter brown . Hypopygium (Figure 8) large, about as high as wide, light honey-colored. Gonocoxite higher than wide, as long as gonostylus, with narrow and dark apical part. Inner side of gonocoxites with sparse and fine setae, intergonocoxal membrane wide and bare. The upper half of the gonocoxites with two symmetrically arranged groups of long bristles almost touching in the middle of the genitals. Gonostylus long-curved oval, with a thin base and broadened apex and long bristles on the outer side. Gonostylus inside slightly bulbous with wide dark subapical lobe covered with short dense bristles.

Ecology. In Ukraine, it is registered in forestry and meadowy habitats. All findings regard the following habitat types: mountain spruce forests on poor soils; acidophilous mesic and moist Scots pine forests; and wet eutrophic and mesotrophic hay grasslands.

Phenology. June–August.

### 3.3. Key to Species of Sciara Registered in Europe

**Figure 9:**
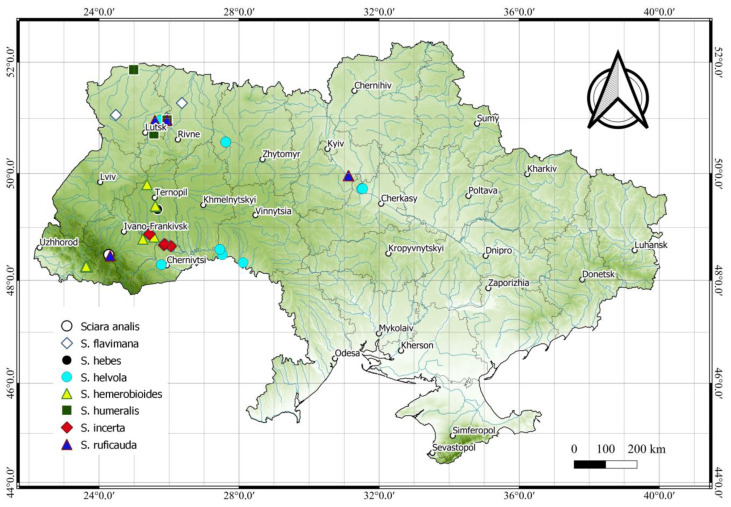
Distribution of *Sciara* species in Ukraine.

According to the results of our research, four species *Sciara flavimana*, *S. hebes*, *S. helvola* and *S. incerta* are recorded for the first time in Ukraine. New localities are given for *Sciara analis*, *S. hemerobioides*, *S. humeralis* and *S. ruficauda* (Figure 9). *Sciara militaris* is not present in our collections and is only known from the literature for Ukraine [7,16,17]. Previous registrations of this species in the country are based on records of the “armyworm” migration phenomenon, but are not confirmed by examination of male terminalia. *Sciara militaris* is registered in Austria, Canada (Alberta), Czech Republic, France (Corsica), Germany, Norway, Poland, Slovakia, Sweden, the United Kingdom (Scotland), USA (Alaska) [8,32,43] and is most likely present in Ukraine.

Thus, nine species of *Sciara* are recorded in Ukraine, but due to the poor study of the sciarid fauna in the country, the presence of all twelve European species is possible. Therefore, in the present key, we have included not only the nine species registered in Ukraine, but also the other three species known in Europe and likely to be found here in the future. Namely, *S. bryophila*, known from Estonia and Finland [9,44], *S. lackschewitzi*, registered in the Czech Republic, Finland, Latvia, Norway and the United Kingdom [8,32,45] and *S. melanostyla*, found in the Russian Federation (European part) and Japan (Honshu) [8,46].
1.Gonostyles compressed and triangular, outer side of apex without dense group of spines, inner side with two lobes (saber-like or bulbous)........................................... 2 – Gonostyles longish, bean-shaped or short and club-like, outer side of apex with or without dense group of spines, inner side with one lobe (if with apical strong and subapical small lobes, then gonoslyles longish and narrow and gonocoxites with two lines of long bristles on the inner margin).................................................... 32.Gonostyles with apical saber-like lobe and baso-medial bulbous lobe (Figure 6)....................................................................................... S. humeralis Zetterstedt, 1851 – Gonostyles without apical saber-like lobe, but with strong subapical lobe and smaller medial lobe.................... S. bryophila Menzel, Salmela and Vilkamaa, 20203.Gonostyles longish and relatively narrow (if long-curved oval, then gonocoxites with two lines of long bristles on the inner margin).................................................... 4 – Gonostyles compact, oval to spherical or bean-shaped, inner margin of gonocoxites without a distinct group of bristles (long and converging or short), with only short setae or bare.......................................................................................... 64.Gonocoxites with two linear groups of long bristles on the inner margin............... 5 – Gonocoxites without a distinct group of bristles (Figure 2)....................................................................................... S. flavimana Zetterstedt, 18515.Gonostyles long-curved oval, inside slightly bulbous with wide dark subapical lobe, without spines; groups of bristles cover at most a half of gonocoxites (Figure 8)............................................................................................. S. ruficauda Meigen, 1818 – Gonostyles longish and relatively narrow, with apical strong and subapical small lobes and spines on the apex; groups of bristles cover more than a half of gonocoxites........................................ S. melanostyla Mohrig and Krivosheina, 19906.Antennae brown or dark brown; all antennal segments concolorous; terminalia dark.................................................................................................................................... 7 – Antennae yellow or light brown; scape, pedicel and base of the first flagellomere yellow, lighter than other flagellomeres; terminalia light-brown to yellow (Figure 4).............................................................................................. S. helvola Winnertz, 18677.Flagellomeres short, length/width of fourth flagellomere body ≤ 2.5....................... 8 – Flagellomeres long, length/width of fourth flagellomere body > 2.5..................... 98.Large species, wing length > 3.0 mm; gonostylus club-like, with bulbous lobe, apical group of spines, several subapical spines and spines under the lobe (Figure 1).................................................................................................... S. analis Schiner, 1864 – Medium-sized species, wing length ≤ 3.0 mm; gonostylus oval, with elongated lobe, two–four apical spines and two additional spines among lobe bristles, but without spines under the lobe.............................................. S. militaris Nowicki, 18689.Gonostylus bean-shaped, with a broadly rounded lobe near the tip..................... 10 – Gonostylus club-like, with subapical wide triangular or thin bulbous lobe...... 1110.Gonostylus with dorsal group of three–six long black spines on projecting hump located inside at the base of the ventral lobe (Figure 3).............. S. hebes (Loew, 1869) – Gonostylus without dorsal group of spines, with only lobe on the tip............................................................................. S. lackschewitzi (Lengersdorf, 1934)11.Wing membrane with macrotrichia at least on apical part; gonostylus with subapical wide tongue-shaped, triangular lobe (Figure 5); large species, wing length is 3.9–4.4 mm…................................................... S. hemerobioides (Scopoli, 1763) – Wing membrane without macrotrichia; gonostylus with subapical thin bulbous lobe (Figure 7); middle-sized species, wing length is 2.6–2.9 mm.............................................................................................. S. incerta Winnertz, 1867

## 4. Discussion

The genus *Sciara* Meigen, 1830 is a relatively large-sized and taxonomically well-defined genus. The genus occurs in all realms (except Antarctica and Subantarctic Islands). In the Holarctic region, *Sciara* is not very species-rich and only 12 species are known from Europe [11,47]. In the Tropical realms, dozens of species are listed [7], but these are mostly unrevised and may include species belonging to other genera. The present study increases the number of *Sciara* species found in Ukraine from five to nine and shows that the Ukrainian fauna are very similar to the Central European fauna. The record of the “armyworm” forming *S. militaris* is known only from the literature [7,16,17] and remains somewhat uncertain, as *S. hemerobioides* may also form “armyworms” [7]. These things considered, new faunistic and habitat records are created, the species occurring in Ukraine are redescribed and illustrated and for the first time, an identification key to all European *Sciara* species is presented. Based on the results of the present study, it is possible that, with more intensive collecting, other species of *Sciara* occurring in Europe may be found in Ukraine, except perhaps for *S. bryophila*, which is currently known only from northern parts of Europe, collected in rich fens in Estonia and Finland [9].

## Data Availability

All data presented in this paper are included within the article and are available for use. All occurrences have been registered with the GBIF (https://www.gbif.org/ accessed on 22 April 2023).
